# Accuracy of Femoral Component External Rotation with all Burr Robotic Assisted Total Knee Arthroplasty

**DOI:** 10.5704/MOJ.2403.003

**Published:** 2024-03

**Authors:** MS Laddha, SV Gowtam, P Jain

**Affiliations:** 1 Department of Orthopaedics, RNH Hospital, Nagpur, India; 2 Department of Arthroplasty and Arthroscocpy, RNH Hospital, Nagpur, India

**Keywords:** total knee arthroplasty, robotic assisted TKA, femoral external rotation, navigated TKA

## Abstract

**Introduction:**

External rotation of femoral component is controversial in Total knee arthroplasty (TKA). The aim of our study is to assess the precision of femoral component external rotation in Robotic Assisted All Burr TKA.

**Materials and methods:**

This is a prospective study of 30 cases who underwent All Burr Robotic Assisted TKA at our institute, RNH hospital. Inclusion criteria were primary and secondary osteoarthritis of the knee and exclusion criteria were revision and partial knee replacement. On Navio robotic system femoral external rotation was planned as per medio-lateral flexion gap balancing and executed with burr. Post-operative CT scan was done in all patients to assess intra-operative planned femoral external rotation.

**Results:**

Out of 30 cases, 20 were female and 10 were male. Mean age was 66.06±7.43 years. On Navio the planned external rotation of femoral component was 2.86±1.16. Average of femoral component external rotation on postoperative CT scan was 3.11±1.16. The mean deviation of achieved femoral component external rotation from planned external rotation was -0.24 to ±0.28. Only 37% patients required 3° external rotation. Correlation between planned and achieved femoral component external rotation was significant, positive and very strong as indicated by r=0.97 and p=0.0001.

**Conclusion:**

All Burr Robotic Assisted TKA provides near accurate femoral component external rotation as planned on Navio with deviation of less than 0.3° between planned and achieved external rotation.

## Introduction

Total knee arthroplasty (TKA) is one of the most successful surgeries1. The success, longevity and excellent functional outcome of total knee arthroplasty is dependent on various factors like accurate component positioning in all three planes, good limb alignment (mechanical or kinematic) and precise ligament balance^2,3^.

Correct femoral component rotation is very important to achieve proper patellar tracking, flexion gap balancing, tibio-femoral stability and limb alignment in flexion^4^. Excessive external rotation of femoral component causes laxity and varus malalignment in flexion^5,6^. Internal rotation of the femoral component causes pain, stiffness, tibio- femoral instability in flexion, patella-femoral instability and failure of the patellar component^7-12^. There is high correlation of patient dissatisfaction, anterior knee pain and implant loosening due to femoral component malrotation^13,14^. Conventional TKA demonstrated femoral component malposition in 15% patients^15^. In TKA correct degree of external rotation of femoral component is debatable but in conventional method 3° femoral external rotation was considered standard as Transepicondyar axis is 3° angled compared to posterior condylar axis. So, any deviation from 3° external rotation was considered as malrotation of femoral component and study by Matziolis *et al*^16^ concluded that conventional TKA had femoral malrotation with a mean deviation of ±2.2°. To improve limb alignment and component placement, computer-aided surgery (CAS) had been introduced since long but studies showed navigated TKA also had femoral component malrotation of 29%^17-18^. Hence achieving correct femoral component rotation is difficult with conventional as well as navigated TKA^19-20^.

Robotic assisted All Burr TKA improved coronal and sagittal placement of component and reduced overall coronal limb alignment outlier from 3° to less than 1.24° and component malposition to less than 1° in sagittal plane^21^. A meta-analysis study by Zhang *et al*^22^ also concluded that robotic assisted TKA increases the accuracy of component positioning^22^.

So, the aim of our study is to assess the precision of femoral component external rotation in Robotic Assisted All Burr TKA.

## Materials and Methods

It was a prospective study of 30 cases who underwent All Burr Robotic Assisted TKA at RNH hospital from July 2021 to November 2021 for grade IV osteoarthritis of knee with moderate to severe varus deformity in all cases without bone loss. Mean age of patient was 66.06 years. Seventeen were right and 13 were left sided. All the patients were operated by the same senior surgeon at our hospital using the All Burr Robotic Assisted Navio system. The inclusion criteria were primary and secondary osteoarthritis of the knee and exclusion criteria were revision knee replacement. Pre-operatively, all cases underwent standard anteroposterior and lateral knee radiographs along with weight bearing anteroposterior scanogram.

**Fig 1:**
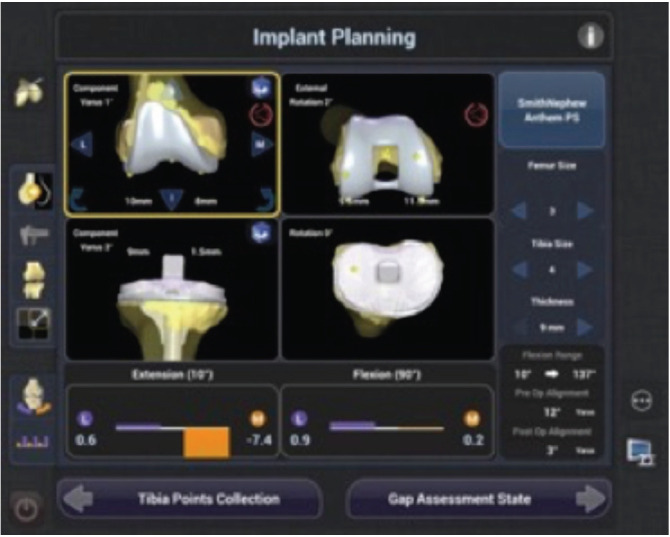
Shows planning of external rotation of the femoral component as per mediolateral gap balancing in flexion and avoiding femoral notching (original photograph from robotic screen during surgery).

All cases were operated using Navio Robotic-Assisted surgical system which involves handheld robotics with an intuitive CT-free registration and patient-specific planning processor^23^. The component placement planning is done as per intra-operative deformity and ligament balancing shown on screen before bone cuts. The external rotation of the femoral component was planned as per medio-lateral gap balancing in flexion and by avoiding femoral notching (Fig. 1). External rotation was planned with reference to the Whiteside line and the Transepicondylar axis.

**Fig 2:**
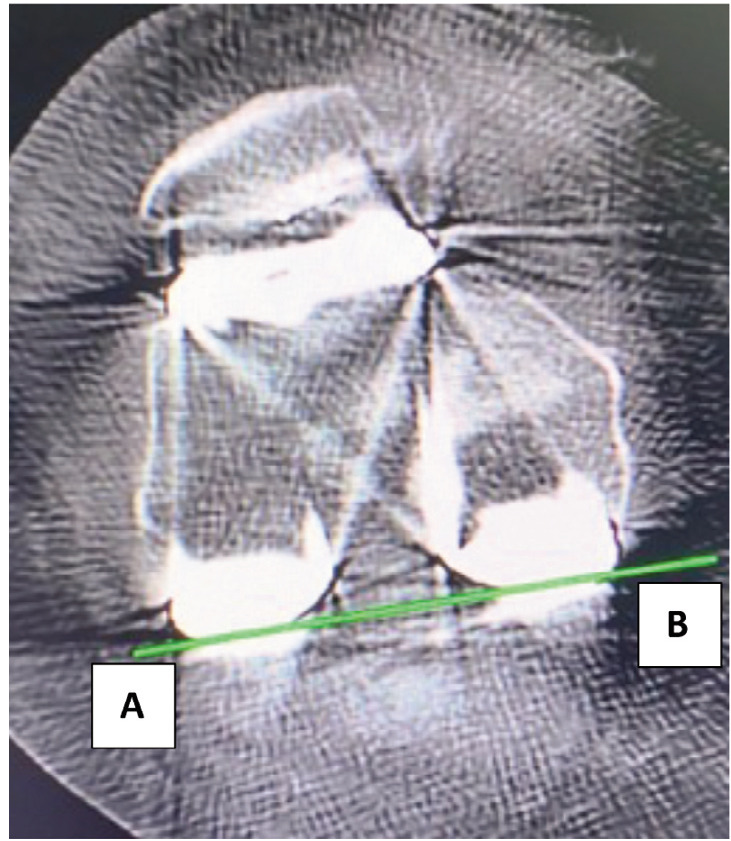
Shows a line AB passing through the distal most point of the femoral component known as femoral prosthesis posterior condylar line (FPPCL).

**Fig 3:**
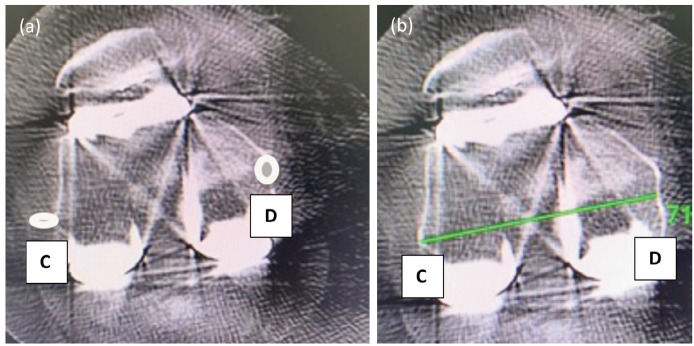
(a) Point C is lateral epicondyle of femur and point D is base of medial sulcus (centre of medial epicondyle). (b) Shows a line CD a line joining the base of medial sulcus (centre of medial epicondyle) and lateral epicondyle known as radiological transepicondylar axis rTEA.

**Fig 4:**
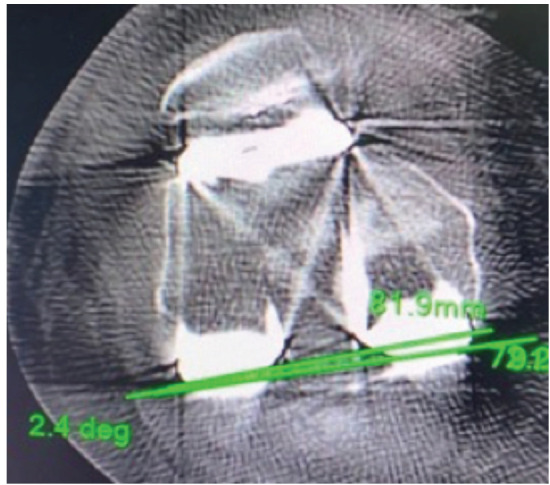
Shows the angle between rTEA (CD) and FPPCL (AB) which is the degree of external rotation of femoral component.

Post-operatively CT scan (Philips incisive 128 slice detectors with OMAR orthopaedic metal artefact reduction) was done within 6 weeks of surgery. An axial 1mm section of CT image of the distal femur which showed clear lateral epicondyle and medial sulcus was used for measurement. Two different radiologists who were unaware of surgical details measured the femoral component external rotation on CT scan and average of the two readings was taken. The rotation of the femoral component was measured by the angle between femoral prosthesis posterior condylar line (FPPCL) and radiographic or "true" TEA (rTEA)^24^. The FPPCL is a line passing through the distal most point of the femoral component (Fig. 2). The rTEA is a line joining the base of medial sulcus (centre of medial epicondyle) and lateral epicondyle identified by its prominent appearance (Fig. 3). The angle between these lines is calculated (Fig. 4). The deviation of post-operatively achieved external rotation from the pre-operatively planned external rotation was calculated and analysed.

## Results

Total number of cases were 30, out of which 20 (66.67%) were female and 10 (33.33%) were male. The number of females were more but there was no significant difference in their age distribution (p=0.45). The mean age was 66.06±7.43 years with 17 (56.67%) right knees were and 13 (43.33%) were left sided but there was no significant correlation with the side (p=0.60).

**Fig 5:**
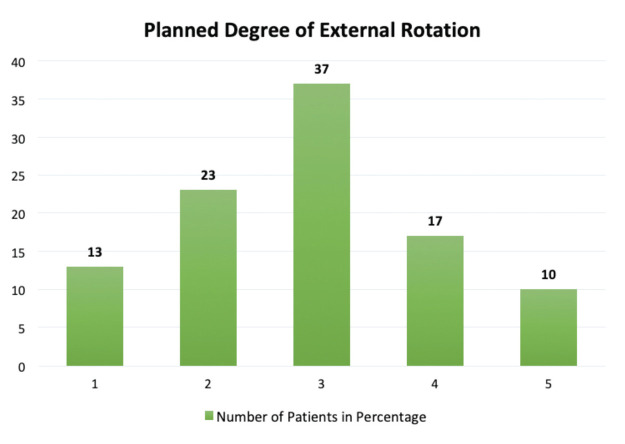
Frequency of planned rotations in degrees.

**Fig 6:**
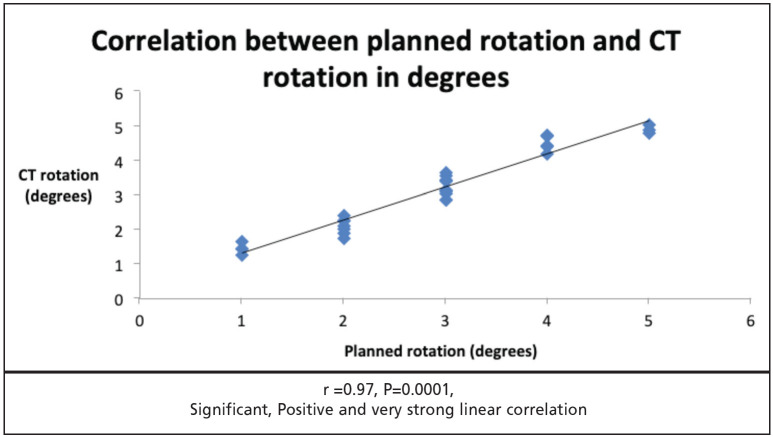
Correlation between planned rotation and CT rotation in degrees.

Post-operative External rotation of femoral component is measured on CT scan as an angle between posterior condylar axis and surgical trans-epicondylar axis. We also used the same method to calculate post-operative femoral component external rotation. We measured the femoral component placement accuracy, by calculating the deviation of femoral component external rotation on CT scan from planned external rotation on Navio system. If planned and achieved femoral component external rotation differ by more than 1° then it would be considered as malrotation. The mean planned external rotation was 2.86±1.16. The mean femoral component rotation on CT scan by first radiologist was 3.13±1.23 and second radiologist was 3.08±1.16. Average of the two readings was 3.11±1.16. The mean deviation of achieved femoral component external rotation from planned external rotation was -0.24±0.28 (Table I). Only 37% cases required 3° external rotation (Fig. 5). Correlation between planned and achieved femoral component external rotation was significant, positive and very strong linear as indicated by r=0.97 and p=0.0001 (Fig. 6).

**Table I:** Statistics summary of intra-operative and post-operative femoral component rotation.

Variable	Cases	Mean	Std.Dev.	Min	Max
Planned	30	2.86	1.16	1	5
Achieved	30	3.11	1.16	1.25	5.05

## Discussion

Soft tissue balance and precise femoral and tibial component placement have been shown to be the main elements contributing to the success of TKA^25,26^. TKA is a very successful surgery, but component malposition is a major worry for aseptic loosening, instability, patient discontent, etc^27^.

According to a study by Chalmers *et al*^28^, femoral component alignment was significantly off when the femoral component was fixed 3° externally to the posterior condylar axis. In a similar vein, Choi *et al*^29^ came to the conclusion that postoperative difference in external rotation of the femoral component did not offer any benefits over external rotation determined intra-operatively. Therefore, the degree of external femoral component rotation after TKA is debatable. Both excessive external and internal femoral component rotation will have a negative impact on the functional outcome of TKA^5,6,8^. Post-operative External Rotation of the femoral component is measured on CT scan as an angle between posterior condylar axis and surgical trans-epicondylar axis. We also used the same method to calculate post-operative femoral component external rotation. We measured the femoral component placement accuracy, by calculating the deviation of femoral component external rotation on CT scan from planned external rotation on Navio system. If planned and achieved femoral component external rotation differ by more than 1° then it would be considered as malrotation.

Laxity in flexion and varus malalignment in flexion^5,6^ are caused by excessive external rotation, while discomfort and valgus malalignment in flexion^7-12^ are caused by excessive internal rotation, patella-femoral instability, and failure of the patellar component. Therefore, we finalised the femoral component external rotation in line with the medial and lateral gap balance in flexion in our robotic aided TKA planning. It ranged from 0° to 5° in our study, and 63% of patients didn't have 3° external rotation. Therefore, 3° femoral component external rotation cannot be considered as the standard that is usually used in conventional TKA.

A total of 15% of instances with conventional TKA had femoral component malrotation, with a mean deviation of 2.2°^17^. Even navigation guided TKAs exhibited femoral component malrotation in 29% of instances. According to the research by Sharkey *et al* on revision TKA, component malposition occurred in 11.8% of revision operations and caused TKA^30^ to fail. The mean deviation of the femoral component's external rotation in our investigation was found to be 0.280, which is quite close to the external rotation that was intended.

Femoral rotational alignment deviation was found to range from 0.02 to 1.15° (mean: 0.52) in studies by Moon *et al*^31^ and from the epicondylar axis in studies by Khuangsirikul *et al*^32^. Clark *et al* study^33^ demonstrated that the All Burr Robotic Aided TKA had a lower cutting error than the computer assisted navigation method. In comparison to oscillating saws, high-speed milling cutters mounted on robot-assisted arms offer superior cutting angular accuracy, which improves the accuracy of femoral component placement^34,35^. Robotic aided TKA achieves the exact external rotation in accordance with the plan in our study since the deviation from the planned external rotation was only about 0.3°.

The limitation of our study is whether this precise femoral component external rotation helps in functional outcome is not assessed. Second limitation is that our study is that it doesn’t have the comparison to the conventional or navigated TKA.

## Conclusion

The All Burr Robotic Assisted TKA provides near accurate femoral component external rotation as planned on Navio with the deviation of less than 0.3° between planned and achieved external rotation.
